# Two Presentations of Neuroglial Heterotopias With Cleft Palate

**DOI:** 10.7759/cureus.89834

**Published:** 2025-08-11

**Authors:** Brenton Stucki, Michelle C Marcincuk

**Affiliations:** 1 Texas College of Osteopathic Medicine, University of North Texas Health Science Center, Fort Worth, USA; 2 Ear, Nose and Throat Center, Cook Children's Medical Center, Fort Worth, USA

**Keywords:** cleft lip/palate, comprehensive otolaryngology, nasopharynx, pediatric airway, surgery

## Abstract

Neuroglial heterotopias, commonly known as nasal gliomas, are rare masses composed of brain tissue located outside the cranial vault. These masses are composed of dysplastic glial cells that have lost their intracranial connections and can present as extranasal, intranasal, or mixed masses. They are thought to result from incomplete closure of the anterior fontanelle between the nasal and frontal bones, which can result in an irregular connection between embryonic ectoderm and neuroectodermal tissue. Although the presentation of a neuroglial heterotopia in a child is uncommon, even more notable is the finding of neuroglial heterotopias visible from a concurrent cleft palate, as this can present additional difficulties in future cleft palate repair and mass excision. Presented in this report are two cases of neuroglial heterotopia found simultaneously with cleft palate. Although these lesions are typically considered benign growths, unmanaged neuroglial heterotopias can result in improper craniofacial development, leading to cosmetic complications and airway obstructions. For accurate diagnosis, thorough histological identification of the embryological tissue origins after surgical biopsy should be performed. Although neuroglial heterotopias and other masses of the nasopharynx, such as teratomas, have similar treatment methods (e.g., surgical resection), detailed histological evaluation of tissue biopsies allows physicians to properly manage cases such as these post-treatment. Early surgical removal of neuroglial heterotopias is encouraged to minimize nasal and craniofacial distortion early in development and to slow or prevent further growth of the lesion. Imaging studies, histological evaluation, surgical procedures, and patient management in the two cases are highlighted in the report to provide physicians with additional insight into possible differential diagnoses and treatment options for physicians with similar case presentations.

## Introduction

Neuroglial heterotopias, commonly known as nasal gliomas, are rare masses of brain tissue located outside the cranial vault. Neuroglial heterotopias typically arise in the midline, as they are thought to arise from failed retraction of herniated neural tissue during early embryogenesis, often through skull base defects such as the foramen cecum [1]. Although these lesions are benign growths of dysplastic cells, neuroglial heterotopias can cause cosmetic and physiological defects, such as the malformation of facial skull bones [1]. While some neuroglial heterotopias are found during routine examinations, most present with symptoms such as nasal obstruction, discharge, or respiratory distress [2,3]. Commonly confused with neuroglial heterotopias are teratomas of the nasal cavity, which are distinct in their origin but exhibit all three embryological tissue origins (i.e., mesodermal, endodermal, and ectodermal) [4]. For accurate diagnosis, histological identification of the embryological tissue origins should be performed. The relationship between neuroglial heterotopias and cleft palate is rooted in the processes of facial development and tissue fusion during embryogenesis. Early surgical removal of gliomas is recommended to minimize nasal and craniofacial distortion early in development and to slow or prevent further growth of the lesion. Here, we present two cases of neuroglial heterotopias presenting in patients with cleft palate in an effort to provide more insight into possible differential diagnoses to physicians with varied case presentations.

This article was previously presented as a poster at the Triological Society 2025 Combined Sections Meeting on January 24, 2025.

## Case presentation

Case 1

**Figure 1:**
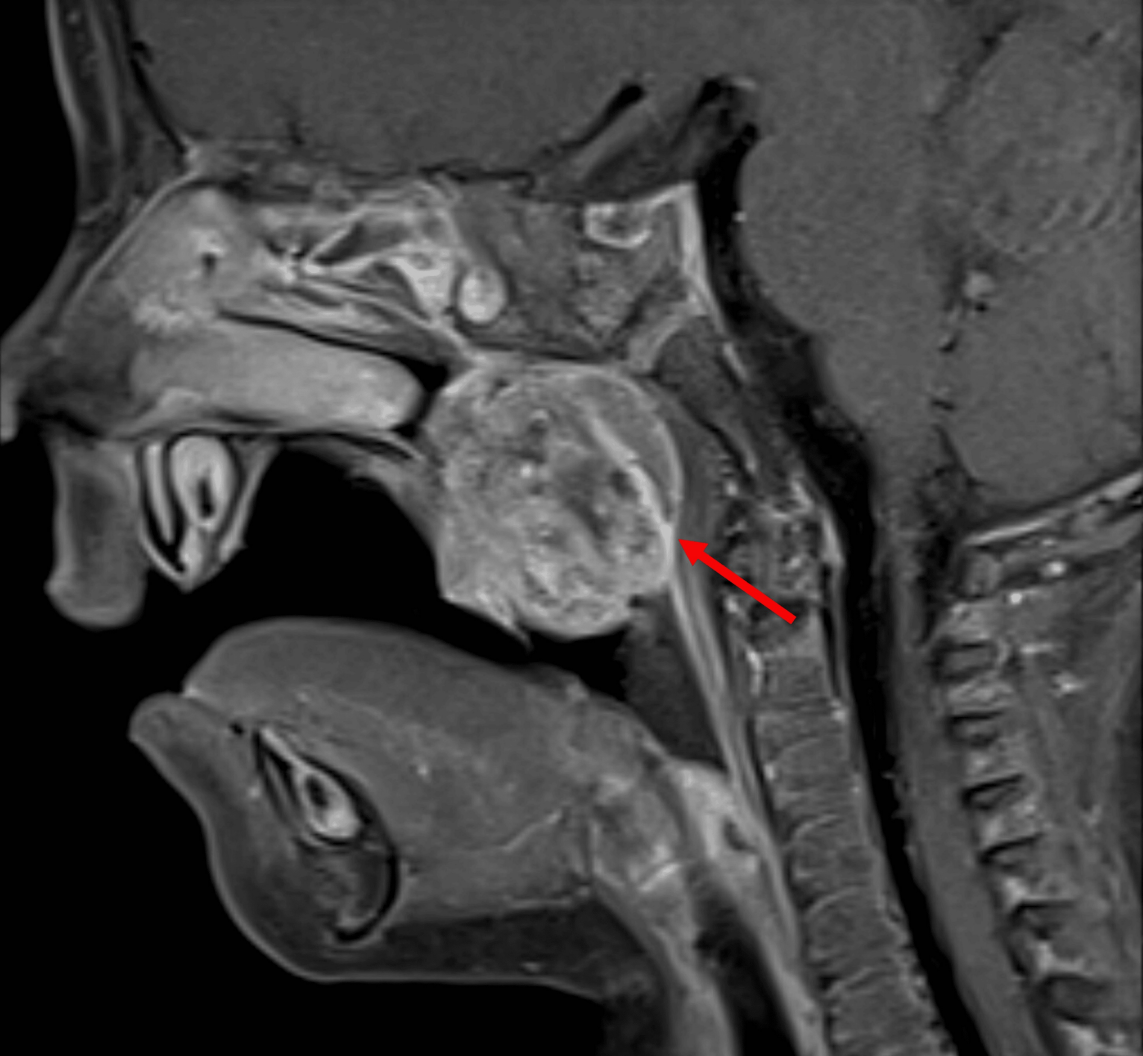
Case 1 MRI Sagittal MRI showing the mass, as highlighted by the red arrow, with a visible lack of communication to the intracranial space.

**Figure 2:**
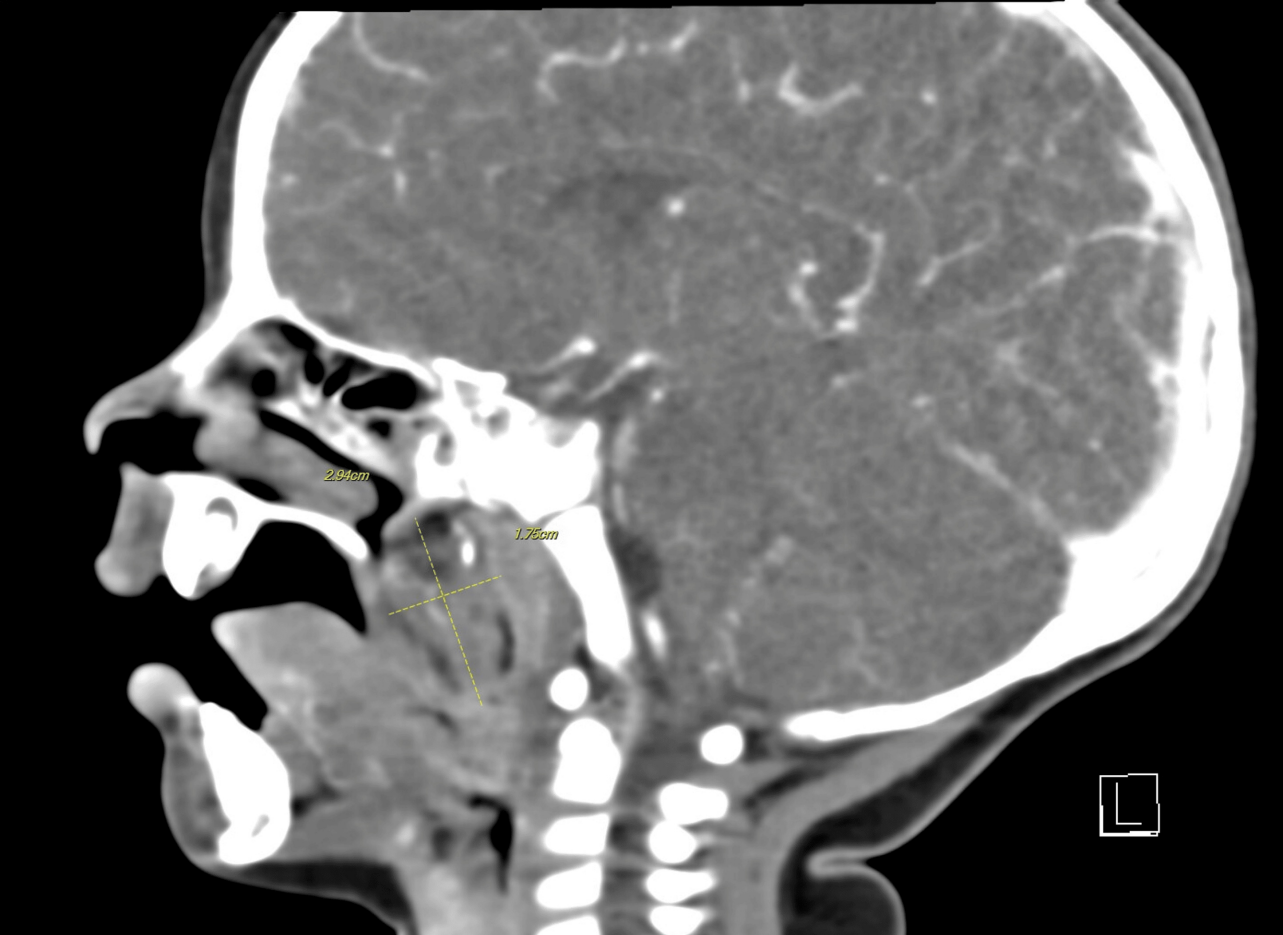
Case 1 CT Preoperative contrast-enhanced sagittal CT showing a midline/right-sided pharyngeal mass with calcifications, fat, soft tissue, and fluid, suggesting a teratoma or epignathus.

**Figure 3:**
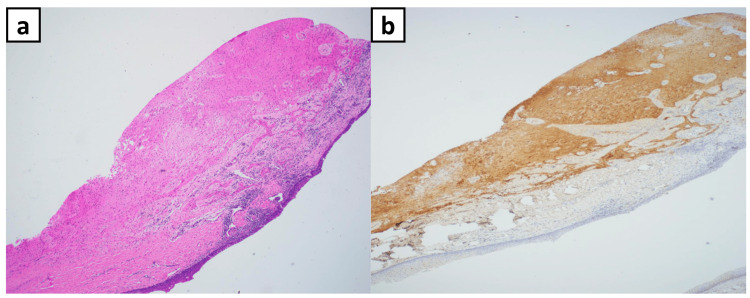
Case 1 Histological Staining of Biopsied Tissue (a) Histological slide of the excised tissue from the mass. (b) A separate histological slide from the same specimen, stained with GFAP, shows positive staining for glial cells.

A 12-month-old female presented to the clinic with a preliminary diagnosis of a palatal mass. On examination, a small mass was visible through the child’s patent cleft palate. Sagittal MRI showed a 2.2 × 1.7 × 2.8 cm (craniocaudal dimensions) midline/right-sided posterior pharyngeal palatal mass with no findings to suggest that the palatal mass had communicated with the intracranial space (Figure 1). A follow-up preoperative contrast-enhanced CT showed a midline/right-sided mass originating from the pharynx, with heterogeneous appearance including small calcifications, fat, soft tissue, and fluid contents (Figure 2). A surgical biopsy of the mass protruding into the cleft palate was then completed. Histology of the biopsy revealed mature glial/neuroglial tissue in a background of salivary gland and lymphoid tissue. A glial fibrillary acidic protein (GFAP) stain was performed on the excised tissue, demonstrating positive staining for glial cells (Figure 3). It was concluded that the mass was predominantly composed of mature neuroglial tissue, with no other convincing teratomatous elements present. Complete surgical resection of the mass was recommended to the patient and completed, along with palatal repair.

Case 2

**Figure 4:**
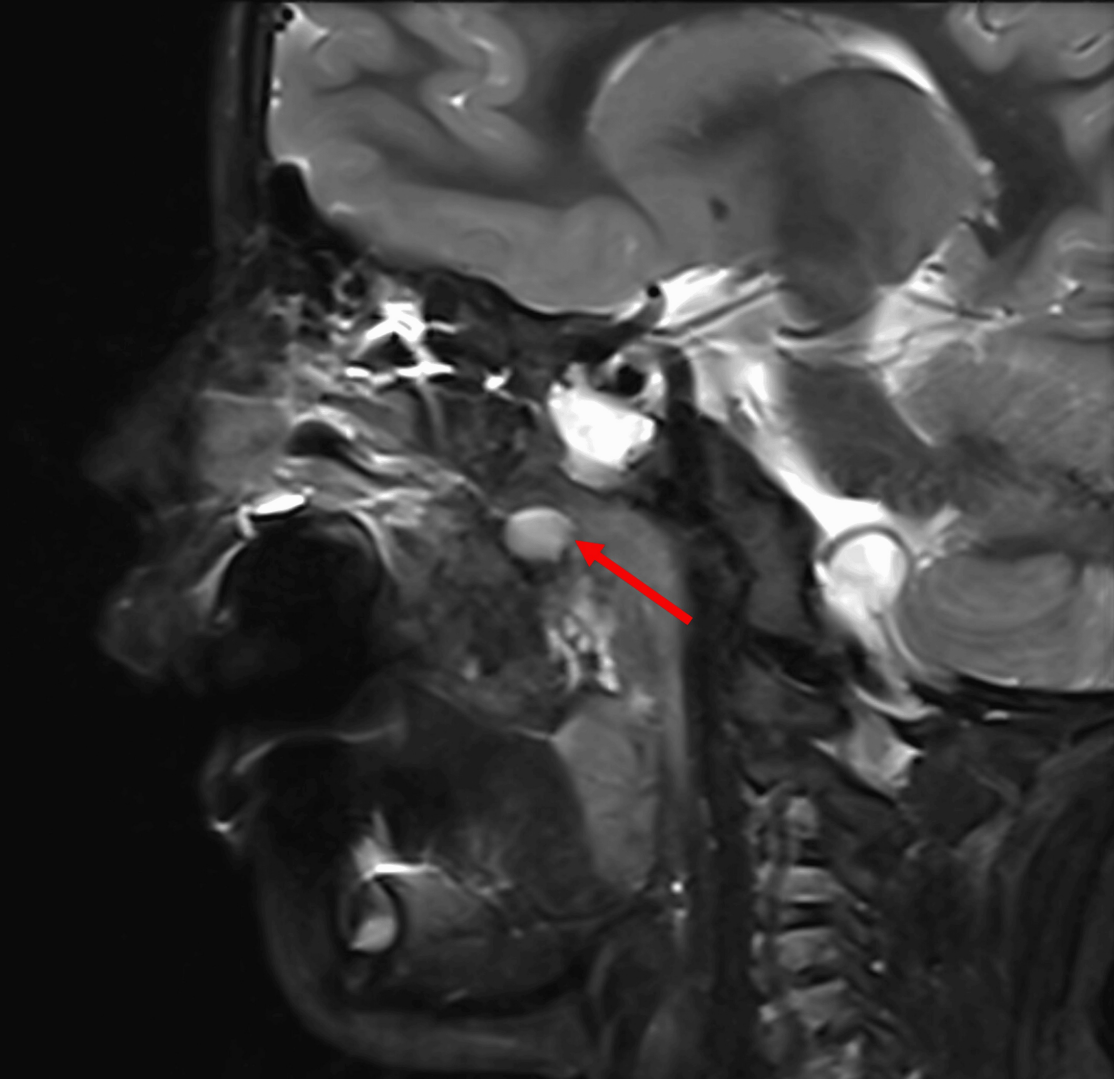
Case 2 MRI Sagittal MRI showing a well-defined oval mass at the right nasopharynx/oropharynx junction, highlighted by the red arrow.

**Figure 5:**
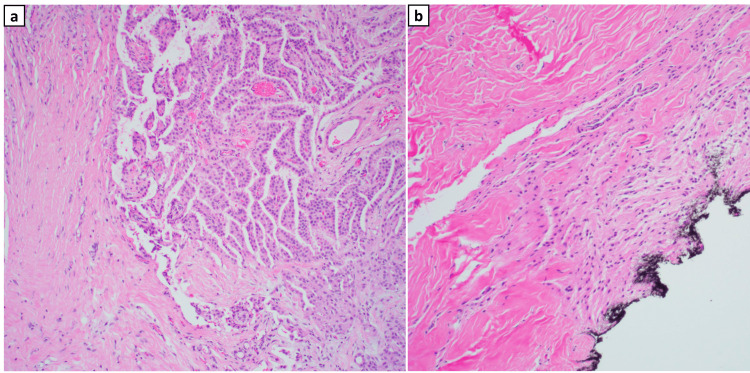
Case 2 Histologic Staining of Biopsied Tissue (a) Suspected teratomatous components in the biopsied tissue, exhibiting origin from the mesoderm, ectoderm, and endoderm. (b) Slide showing mature glial tissue, supporting exclusion of teratoma.

A 21-day-old female with a history of Pierre Robin sequence and tongue-based airway obstruction was referred to the otolaryngology clinic for evaluation of associated craniofacial anomalies. The patient was well-appearing and asymptomatic. Helical CT scanning of the facial bones without intravenous contrast was performed, and multiplanar reformats were completed and reviewed; a left parasagittal cleft in the soft palate was suspected. The patient returned at two years of age, and a follow-up MRI of the face revealed a well-circumscribed, oval-shaped mass in the right lateral aspect of the nasopharynx/oropharynx junction, along with re-demonstration of the cleft palate (Figure 4). The mass measured approximately 2.5 × 1.6 × 2.4 cm. Complete surgical resection was recommended. Complete surgical resection was recommended. Upon histological examination of the frozen biopsies collected during surgery, mesodermal, ectodermal, and endodermal features were observed, and initially, a potential teratoma was suspected. A complete biopsy was completed, and further histological examination was performed. It was concluded that the finding of mesodermal (bone and fat) and endodermal components (respiratory-lined cysts) represented derivatives of mature tissue from the surgical site rather than ectopically located cells (Figure 5). The mass was diagnosed as a neuroglial heterotopia. A successful nasal endoscopy was completed approximately two years after the completion of the surgery, and no mass recurrence was detected.

## Discussion

Neuroglial heterotopias are not true neoplasms and have no communication with the CNS. They are composed of dysplastic glial cells that have lost their intracranial connections and can present as extranasal, intranasal, or mixed masses [5]. They are thought to result from incomplete closure of the anterior fontanelle between the nasal and frontal bones, which can result in an irregular connection between embryonic ectoderm and neuroectodermal tissue [6]. The relationship between neuroglial heterotopia and cleft palate may lie in the processes of facial development and tissue formation during embryogenesis, as both abnormalities are a result of improper closure of the midline structures during development [1,7]. The development of a palatal cleft typically occurs between the seventh and twelfth week of embryonic development, when the palatal shelves grow out from the maxillary prominences and reorient, fusing at the midline to separate the oral and nasal cavities [8]. When these structures fail to merge correctly, a gap in the hard and/or soft palate may occur, resulting in a cleft palate. Given that both conditions involve disruptions in midline fusion in utero, their co-occurrence may suggest a shared embryologic framework [9].

Histological examination is vital in confirming the presence of neuroglial heterotopias. In one case, mature glial/neuroglial tissue was identified amidst native salivary gland and lymphoid tissue. Initially, teratomatous components were suspected in the second case, but further review revealed that these components were native to the surgical site. GFAP stains were performed in both cases, aiding in the diagnostic process. While the initial treatment for teratomas and neuroglial heterotopias may be similar, the recurrence rates and behavior of tissue growth differ [10,11], emphasizing the importance of an accurate diagnosis. Highlighted in these cases was the need for distinction between the two congenital lesions. Although both masses share the characteristic of disorganized or misplaced tissue in the nasal cavity, their histopathology and embryologic origins remain different. Unlike encephaloceles, neuroglial heterotopias lack intracranial communication, and unlike teratomas, they do not contain multiple germ layers [5]. In the cases described in this report, immunohistochemical staining played a pivotal role in confirming the final diagnosis, in addition to radiologic imaging (including CT with confirmatory MRI) to rule out CNS communication [12].

Despite their benign nature, neuroglial heterotopias can cause significant morbidity in affected patients if not properly treated. Namely, these masses have been associated with airway obstruction, craniofacial distortion, and feeding difficulties [2,13]. To reduce these risks, early surgical intervention is recommended. These cases highlight the importance of surgical excision as the primary approach to managing neuroglial heterotopias. Following surgical biopsy in both cases, complete resection with negative margins was performed, later followed by palatal repair. At present, there is no direct evidence suggesting that nasal gliomas should be excised specifically at the time of palate repair. However, combining surgeries could be considered to minimize the number of anesthetic exposures and surgical interventions a child undergoes. Failure to perform proper excision of nasal gliomas can lead to recurrence rates as high as 30% [11].

## Conclusions

These two case presentations emphasize the significance of early recognition and appropriate management of neuroglial heterotopias in pediatric patients, especially those with cleft palate. Timely detection and surgical intervention were paramount for achieving favorable outcomes. The aim of presenting such cases is to provide more insight into possible differential diagnoses and treatment options for physicians with similar case presentations.
